# Adoption of the Wet Surface Treatment Technique for the Improvement of Device Performance of Enhancement-Mode AlGaN/GaN MOSHEMTs for Millimeter-Wave Applications

**DOI:** 10.3390/ma14216558

**Published:** 2021-11-01

**Authors:** Chun Wang, Yu-Chiao Chen, Heng-Tung Hsu, Yi-Fan Tsao, Yueh-Chin Lin, Chang-Fu Dee, Edward-Yi Chang

**Affiliations:** 1 Department of Material Science Engineering, National Yang Ming Chiao Tung University, 1001 Tah Hsueh Road, Hsinchu 30010, Taiwan; wangben2920.mse05g@nctu.edu.tw (C.W.); nctulin@yahoo.com.tw (Y.-C.L.); 2 Department of Electrical Engineering, National Yang Ming Chiao Tung University, 1001 Tah Hsueh Road, Hsinchu 30010, Taiwan; bigjj600@gmail.com; 3 International College of Semiconductor Technology, National Yang Ming Chiao Tung University, 1001 Tah Hsueh Road, Hsinchu 30010, Taiwan; hthsu@nctu.edu.tw (H.-T.H.); elle1352.05g@g2.nctu.edu.tw (Y.-F.T.); 4 Institute of Microengineering and Nanoelectronics (IMEN) Level 4, Research Complex, University Kebangsaan Malaysia, Bangi 43600, Malaysia; cfdee@ukm.edu.my

**Keywords:** GaN, HEMT, E-mode, wet surface treatment, power performance, millimeter-wave applications

## Abstract

In this work, a low-power plasma oxidation surface treatment followed by Al_2_O_3_ gate dielectric deposition technique is adopted to improve device performance of the enhancement-mode (E-mode) AlGaN/GaN metal-oxide-semiconductor high-electron-mobility transistors (MOSHEMTs) intended for applications at millimeter-wave frequencies. The fabricated device exhibited a threshold voltage (V_th_) of 0.13 V and a maximum transconductance (g_m_) of 484 (mS/mm). At 38 GHz, an output power density of 3.22 W/mm with a power-added efficiency (PAE) of 34.83% were achieved. Such superior performance was mainly attributed to the high-quality Al_2_O_3_ layer with a smooth surface which also suppressed the current collapse phenomenon.

## 1. Introduction

The quest for a high-capacity 5G network for increased data transmission requires wide bandwidth and high signal-to-noise ratio (SNR) to maintain the quality of service (QoS) of the system. Since the spectrum below 20 GHz has been crowded with commercial applications, allocation of bandwidth wide enough to accommodate the required channel capacity is almost impossible. As a result, operating at millimeter-wave frequencies has been the only solution for next-generation communication systems targeting at multi-giga-bit data transmission with minimum latency. In comparison with Si- and GaAs-based transistors, GaN-based HEMTs exhibit superior performance at high frequencies in terms of power density and efficiency due to the characteristics of the wide bandgap, high electron mobility and large breakdown field of GaN material. Successful demonstrations of the depletion-mode (D-mode) devices have been reported in [1,2,3].

Other than the D-mode devices, the E-mode devices exhibit certain advantages in terms of practical operations at system level. Since the devices require only positive supply, the complexity of the biasing network can greatly be reduced. Moreover, elimination of the negative supply also helps the suppression of the system noise. In the past, several approaches were reported for the development of E-mode devices. Fluorine plasma treatment was applied to insert negative charges into the barrier layer, resulting in a positive shift with the threshold voltage as a depletion region was formed in the 2DEG channel. A maximum output power density of 3.6 W/mm and peak PAE of 42% were measured at 18 GHz from the E-mode AlGaN/GaN heterojunction FET (HFET) [4]. However, the current collapse is a big issue caused by the plasma implantation. The technique of applying a p-type GaN layer under the gate region can help avoiding the damage caused by plasma. Normally-off HEMTs with the formation of the p-n junction exhibited the cut-off frequency (f_T_) and maximum oscillation frequency (f_max_) of 6.0 and 9.8 GHz by adopting a gate-all-around structure in [5].

Gate recess is the major technology for the realization of the E-mode devices [6,7,8]. The barrier thickness under 8 nm with a proper gate-recessed process exhibited excellent RF characteristics in terms of the f_T_ and f_max_ in [8]. The major issue with a gate recess process is the unavoidable damage to the surface leading to possible performance degradations. Thus, techniques of surface treatment, damage-free gate recess, and dielectric passivation have been proposed to suppress the possible damage that causes the current collapse phenomenon [9,10,11,12,13,14,15]. There are various surface treatments and cleaning methods, such as UV/ozone [16], wet chemical [17], plasma [18], descum [19] and O_2_ treatment [20] which have been adopted for the improvement of the surface states. For instance, with the SF_6_ plasma treatment, the gate leakage and pulse I–V characteristics were improved effectively due to the reduction in the amount of carbon on the semiconductor surface [18]. Besides, an Al_2_O_3_ dielectric layer under the gate region was adopted to suppress the hydrogen-induced weak bonds for leakage current reduction in [11]. The fabricated E-mode metal-insulator-semiconductor HEMTs (MISHEMTs) demonstrated an output power density of 5.76 W/mm and a PAE of 57% at 4 GHz.

In this work, we fabricated an enhancement-mode AlGaN/GaN metal-oxide-semiconductor high-electron-mobility transistor (MOSHEMT) using the wet surface treatment technology followed by the deposition of a high-quality Al_2_O_3_ layer. Compared with hydrochloric acid cleaning, a better surface morphology was achieved after the adoption of the wet surface treatment. Moreover, the imperfect layer of the gate region surface which included the native oxide and the bombardment damage caused by the plasma etching process could be effectively removed. Improvement in the device performance was observed with such surface treatment due to the suppression of the current collapse phenomenon. The maximum output power density and peak PAE were measured to be 3.22 W/mm and 34.83%, respectively, at 38 GHz.

## 2. Materials and Methods

**Figure 1:**
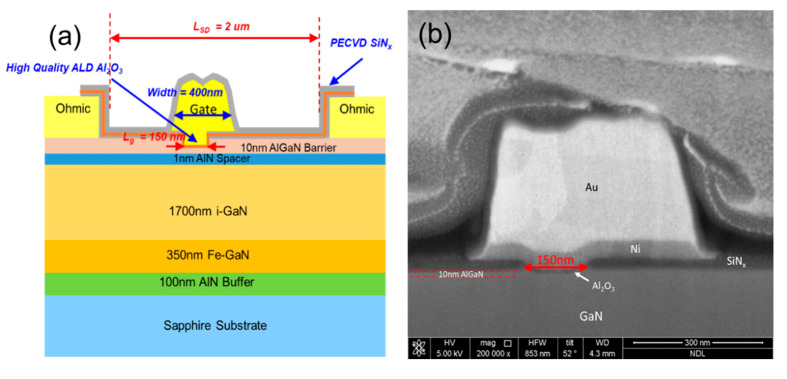
(**a**) Epitaxial structure and (**b**) scanning electron microscopy (SEM) image of the T-shape gate for the thin barrier AlGaN/GaN metal-oxide-semiconductor high-electron-mobility transistor (MOSHEMT).

**Figure 2:**
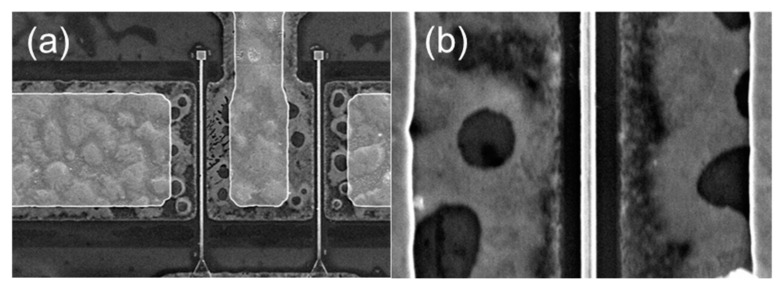
(**a**) Scanning electron microscope (SEM) image of top view of the high-electron-mobility transistor (HEMT) device and (**b**) SEM image of the magnification of the Source/Drain spacing region.

**Figure 3:**
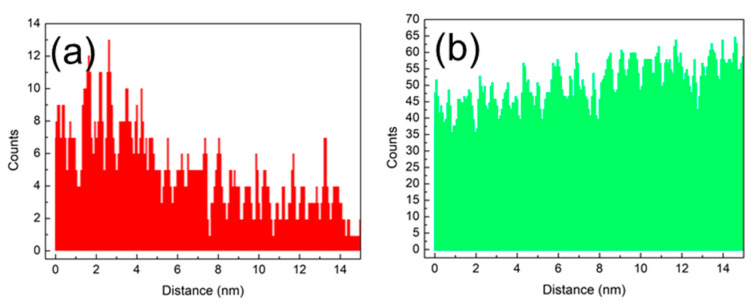
Energy-dispersive X-ray spectroscopy (EDX) analysis of (**a**) aluminum and (**b**) gallium element.

**Figure 4:**
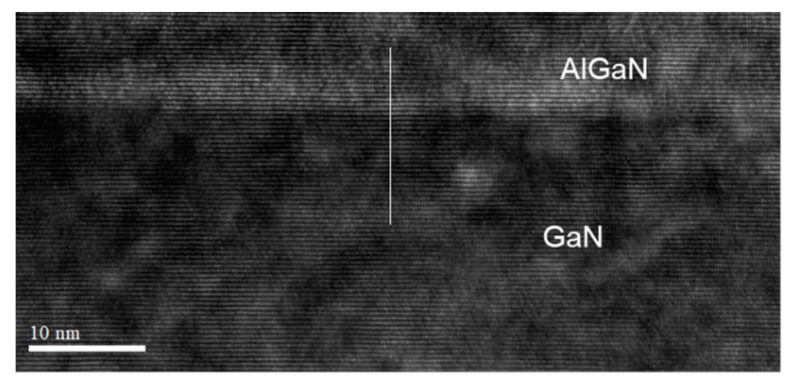
The cross-section high-resolution transmission electron microscopy (HRTEM) image of the interface.

Figure 1 shows the cross section of the thin-barrier AlGaN/GaN MOSHEMT grown on 4-inch Sapphire substrate by metal-organic chemical vapor deposition. The T-gate with asymmetric head toward the drain side was adopted for the purpose of the breakdown-voltage enhancement. Figure 2 displays the top view SEM images of AlGaN/GaN MOSHEMTs at different magnifications. The epitaxial structure consists of a 100 nm AlN nucleation layer, a 350 nm iron-doped GaN buffer layer, a 1700 nm i-GaN layer, a 1 nm AlN spacer layer, and a 10 nm Al_0.26_Ga_0.74_N barrier layer. Room temperature Hall effect measurement exhibits a sheet carrier density of 9.7 × 10^12^ cm^−2^, a sheet resistance of 324 Ω/□, and an electron mobility of 1980 cm^2^/V·s, respectively. Figure 3 shows EDX analysis of aluminum and gallium elements. It is observed that the aluminum was rich in the AlGaN barrier and gradually decreased when it reached the GaN buffer layer. The cross-section HRTEM image is also shown in Figure 4.

**Figure 5:**
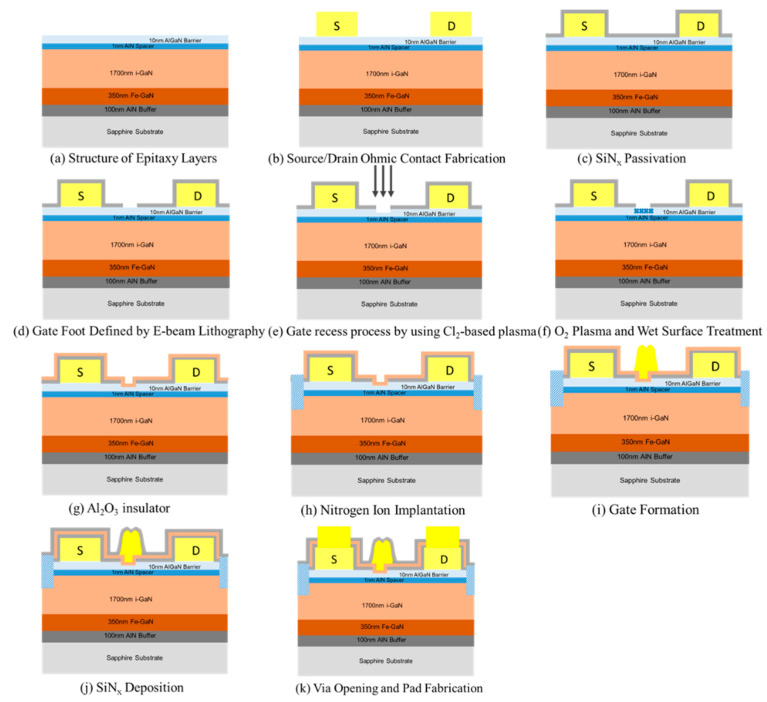
Fabrication process for the thin barrier E-mode AlGaN/GaN MOSHEMT.

Device fabrication started with the wafer cleaning by dipping in the acetone and isopropanol solution to remove the organic particles or contaminations. The ohmic contact consisted of a Ti/Al/Ni/Au metal stack deposited by ULVAC EVA650 E-gun evaporation system (EVA650, Ulvac, Hsinchu, Taiwan). Ohmic metal alloying was then formed at 820 °C for 30 s in N_2_ ambient using a Premtek RTP-T41 rapid thermal annealing (RTA) system. A 25 nm-SiN_x_ was deposited by STS310PC plasma-enhanced chemical vapor deposition (PECVD) with in-situ nitrogen plasma treatment prior to the film deposition [21]. The SiN_x_ film could reduce the dangling bond on the surface and act as hard mask at the recess region. The 150 nm gate foot was defined by using a 50 kV JEOL e-beam lithography (EBL) system (JBX 6000 FS) and the gate region was opened by fluorine-based ICP dry etching to remove SiN_x_ film. Next, the gate recess process was performed using Cl_2_-based plasma with the following parameters: 40 sccm flow rate, 0.1 Pa pressure, and 200 W of RF power with 5 W of bottom bias. O_2_ plasma was performed by the ICP system (Nesca-20 plus, Cello Technology, Hsinchu, Taiwan) with low RF power of 50 W and the 5 W for the bottom bias. Afterwards, a thin oxide layer was formed on the surface. Then, the oxide layer was removed by wet surface treatment using NH_4_OH solution for 5 min. A 7 nm Al_2_O_3_ insulator was deposited by a Fujitec G2 atomic layer deposition (ALD) system (Fujitec G2, Veeco, Plainview, NYC, USA) immediately after the surface treatment. The growth rate was 1 Å/cycle for 70 cycles and the temperature was kept at 250 °C. Afterwards, the active region was defined by nitrogen ion implantation. With the use of the ZEP/GL-2000 photoresists, the T-shape gate was formed as shown in Figure 1 and the width of the foot and head were 150 nm and 400 nm. A 15 nm SiN_x_ layer was deposited by PECVD after Ni/Au gate metal deposition and the lift-off process. The recessed depth of the gate region was approximately 4 nm. Finally, Ti/Au was deposited as an interconnect metal. The process flow is shown in Figure 5. The gate-source and gate-drain distance were 0.7 μm, and 1.1 μm, respectively.

**Figure 6:**
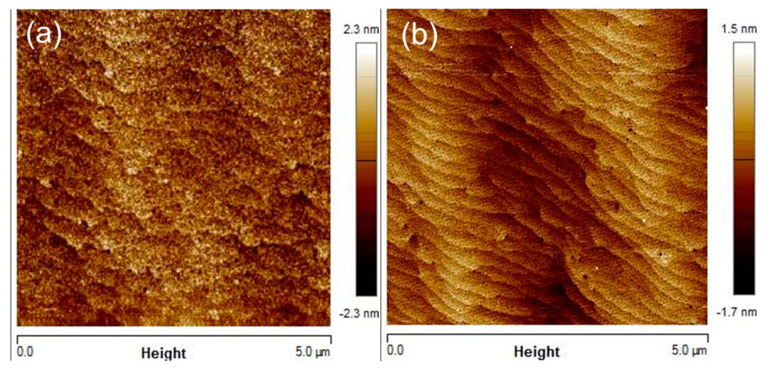
Surface morphology of the AlGaN/GaN heterostructure after etching barrier layer (**a**) without treatment and (**b**) with wet surface treatment.

Figure 6 shows the root mean square roughness of the AlGaN layer surface morphology by an atomic force microscope. Substantial reduction in the surface roughness from 0.66 nm to 0.4 nm was observed for the device with surface treatment. Moreover, an AFM image with an atomically clean morphology was achieved after applying the treatment.

## 3. Results and Discussion

### 3.1. X-ray Photoelectron Spectroscopy (XPS) Measurement

**Figure 7:**
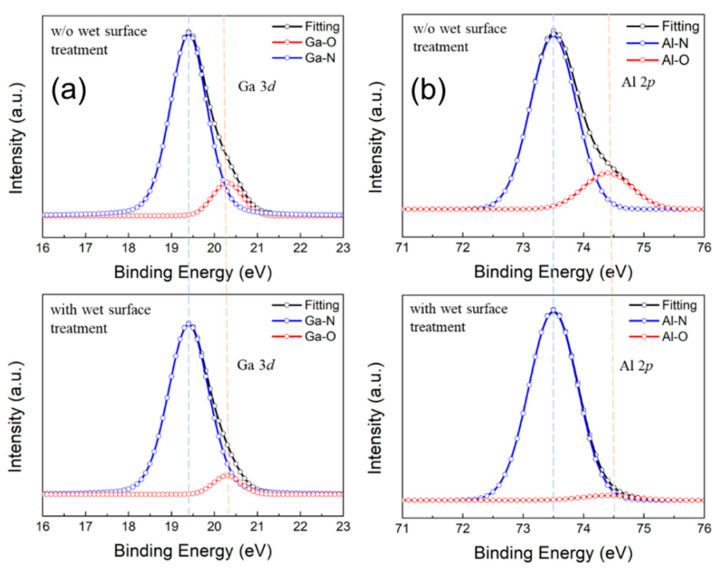
The X-ray photoelectron spectroscopy (XPS): (**a**) Ga 3*d* and (**b**) Al 2*p* core level spectra of the AlGaN surface with and without wet surface treatment.

X-ray photoelectron spectroscopy (XPS) was adopted to investigate the surface chemical composition after applying the wet surface treatment. Generally, an imperfect layer caused by the environment pollutants, the native oxide, and the bombardment damage existed after the gate recess process. The existence of such a layer would degrade the device characteristics. It is thus of crucial importance to address this issue especially for devices at millimeter-wave frequencies. Figure 7 shows the Ga 3*d* and Al 2*p* core-level spectra of the control sample with HCl surface treatment and the sample with wet surface treatment, respectively. The Ga 3*d* and Al 2*p* peaks were separated into four main peaks Ga–O, Ga–N, Al–O and Al–N bonds. It is obvious that the intensity of the Ga–O and Al–O with a larger binding energy are much lower in the experimental sample. In other words, the Ga–O and Al–O bonding proportion decreased at the surface region. Such an effect indicates that with the application of wet surface treatment, the imperfect layer can be effectively removed. In [17], the XPS analysis showed the reduction of the Ga–O and Al–O percentage after the TMAH treatment, which was considered to be the removal of the damage and the oxides at the AlGaN gate surface region.

### 3.2. Direct Current (DC) and Pulsed Current–Voltage (I–V) Characteristics

**Figure 8:**
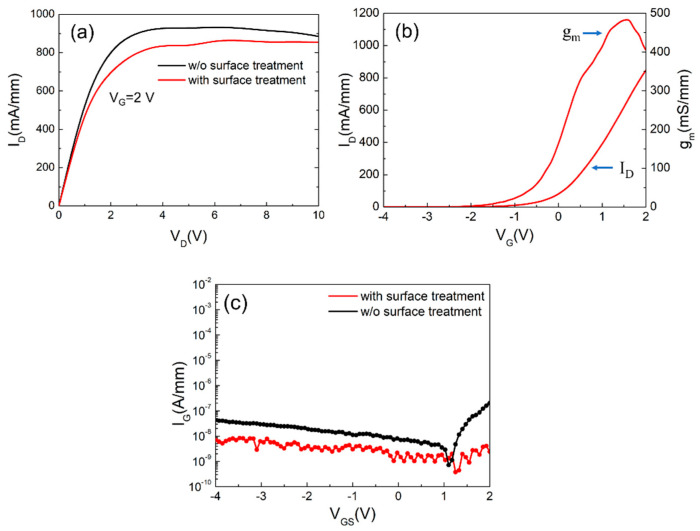
(**a**) The measured drain current as functions of drain voltage for devices with and without surface treatment. (**b**) The measured drain current and extrinsic transconductance (g_m_) with surface treatment. (**c**) Gate leakage of recess gate HEMTs with and without surface treatment.

**Figure 9:**
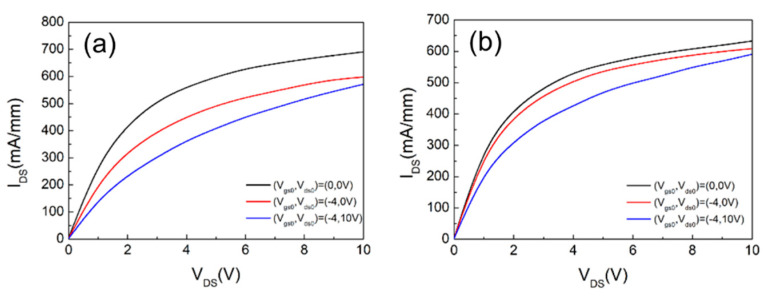
Pulsed I–V characterization with 200-ns pulse width and 0.1% duty cycle for 2 × 25 μm^2^ thin barrier AlGaN/GaN HEMTs (**a**) without and (**b**) with wet surface treatment.

The measured DC characteristics of the thin barrier E-mode AlGaN/GaN HEMTs is shown in Figure 8. As observed, the fabricated 2 × (0.15 × 25) μm^2^ devices with surface treatment exhibits a lower maximum drain current of 859 mA/mm compared to the devices without surface treatment, which has a maximum current of 931 mA/mm at V_G_ = 2 V. After the wet surface treatment, a peak extrinsic transconductance (g_m_) 484 mS/mm and a threshold voltage (V_th_) of +0.13 V were achieved at V_DS_ = 5 V due to the removal of the surface oxidation layer by wet recess. While scaling down the distance between gate and channel, higher g_m_ and lower current density were obtained for E-mode device. As for the gate leakage current for both cases shown in Figure 8c, further improvement in the leakage current is also observed for the device with surface treatment. The forward gate current of the device with wet surface treatment exhibited two orders of magnitude lower than that without surface treatment, evidencing the effect of the removal of the imperfect layer. Further investigation on the trapping levels at the interface was conducted through the pulsed-IV measurements. Figure 9 shows the results with a 200 ns pulse width and 0.1% duty cycle. With the gate stress measurements under a quiescent bias of (V_GS0_, V_DS0_) = (−4 V, 0 V), the device with wet surface treatment shows minor collapse ratio of 3.7% which is much smaller than that of 17.78% for the one without treatment. It is obvious that the plasma damage caused by the dry etching process can be suppressed after the combination of the oxidation and wet etching treatment. The drain lag ratio has also decreased from 17% to 12.58% at V_GS_ = 1 V and V_DS_ = 5 V under the condition (V_GS0_, V_DS0_) = (−4 V, 10 V). Improvement in the current collapse phenomenon has been identified from both drain and gate lad measurements. It is worth mentioning that the drain lag results are owing to the deep traps in epitaxial layers, such as GaN iron-doped buffer [22,23]. These trapping electrons from defects may cause the degradation of the device’s electrical properties.

### 3.3. Small-Signal Characteristics Comparison

**Figure 10:**
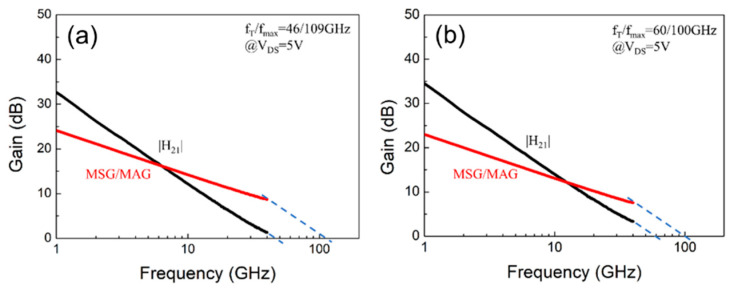
The extracted cut-off frequency (f_T_) and maximum oscillation frequency (f_max_) for thin barrier AlGaN/GaN MOSHEMT devices: (**a**) without and (**b**) with surface treatment.

**Figure 11:**
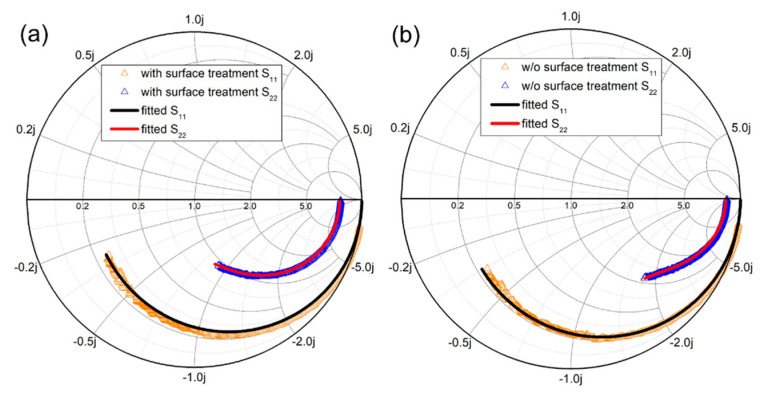
The Smith chart of the measured and fitted curves of the (**a**) with and (**b**) without surface treatment device.

The small-signal RF S-parameters were measured from 100 MHz to 40 GHz using a Keysight N5227B network analyzer. A standard short-open-load-through (SOLT) calibration method was adopted to calibrate the system with the reference planes set at the tips of the corresponding probes. Figure 10 shows the measurement results for the devices with and without wet surface treatment. The extracted unit current-gain cutoff frequency (f_T_) and the maximum oscillation frequency (f_max_) were 46/60 GHz and 109/100 GHz for the device with/without surface treatment. We have used the small-signal equivalent circuit using the procedure in [24] to extract parameters. Figure 11 shows that there is no difference between the Smith chart of the measured and fitted curves. The total gate capacitance (C_gs_ + C_gd_) for devices without and with surface treatment are 98.95 fF and 86.7 fF, respectively. Thus, the higher cut-off frequency (f_T_) of the device with wet surface treatment can be attributed to the lower gate capacitance. However, slight degradation in f_max_ for the device with surface treatment is possibly due to the decrease of the output current and the lower 2DEG density.

### 3.4. Large-Signal Performance

**Figure 12:**
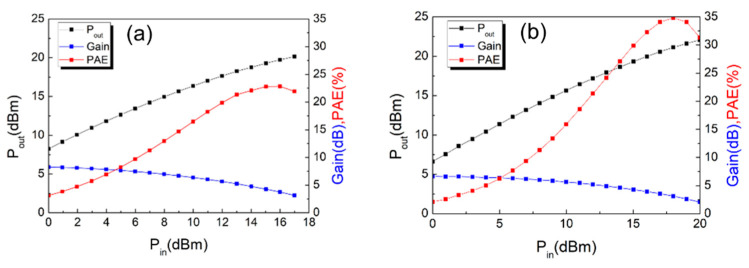
Large signal radiofrequency (RF) output power characteristics of (**a**) without (**b**) with wet surface treatment (V_DS_ = 10 V) at 38 GHz.

The large-signal performance was characterized using an on-wafer load-pull system at 38 GHz under continuous mode excitation. Figure 12 shows the measurement results with the device biased at class AB operation and the impedance is optimized for maximum output power. The optimal source and load impedance for the device without treatment are (32.20 + *j*24.12) Ω and (92.15 + *j*126.12) Ω and those for the device with treatment are (35.85 + *j*28.31) Ω and (226.07 + *j*216.15) Ω, respectively. Under these conditions, a maximum output power density of 2.1 W/mm with a linear power gain (G_p_) of 8.2 dB and a PAE of 22.84% were obtained at V_DS_ = 10 V for the device without wet surface treatment. On the other hand, with the suppression of current collapse, a higher maximum output power density of 3.22 W/mm and a PAE of 34.83% were achieved for the device with surface treatment. Such a substantial improvement in the performance at Ka-band is mainly attributed to the suppression of the current collapse issue [25]. The measurement results have evidenced that the device technology presented in this paper is promising for future applications in power amplifiers at millimeter-wave frequencies. Furthermore, the proposed MOSHEMT outperforms other E-mode AlGaN/GaN HEMTs on SiC substrate [4,7,26]. The reported work exhibits a good power performance at relatively low V_DS_. In all, the experimental results of the thin barrier E-mode AlGaN/GaN HEMT on Sapphire shows a great potential for low power consumption and low-cost power amplifier application.

## 4. Conclusions

A high-performance thin barrier enhancement-mode AlGaN/GaN HEMT was fabricated with wet surface treatment and high-quality Al_2_O_3_ as the gate dielectric layer. After the dry etching process and wet etching treatment, the damaged surface layer was removed. Moreover, the current collapse was eliminated due to the reduced trapping effect. Compared to a device without surface treatment, substantial improvement in the radiofrequency (RF) performance was achieved. The device exhibited a superior power performance, including a maximum output power density of 3.22 W/mm and a peak PAE of 34.83% at 38 GHz. These results demonstrate an attractive technique for fabricating high-performance E-mode HEMTs for power amplifier applications at millimeter-wave frequencies.

## Data Availability

The data presented in this paper are available on request from the corresponding author.
